# Laser biospeckle as a method to investigate the short-term effects of far-red light on an arugula (*Eruca sativa* Mill) plant

**DOI:** 10.3389/fpls.2025.1496790

**Published:** 2025-02-25

**Authors:** Hibiki Igarashi, Takeshi Baba, Kairi Takemura, Takahiro Kono, Hirofumi Kadono, Jun Yamada, Uma Maheswari Rajagopalan

**Affiliations:** ^1^ Department of Mechanical Engineering, Shibaura Institute of Technology, Tokyo, Japan; ^2^ Shibaura Institute of Technology, Tokyo, Japan; ^3^ Tokyo Metropolitan University, Hino, Japan; ^4^ Graduate School of Science and Engineering, Saitama University, Saitama, Japan; ^5^ Innovative Global Program, Shibaura Institute of Technology, Tokyo, Japan

**Keywords:** laser, biospeckle, plant development, photoreceptor, short-term effects, far-red

## Abstract

With rapid climate change, it has been increasingly difficult to grow different crops, and as an alternative method, artificial cultivation in controlled environments has evolved into a new sustainable agriculture practice. However, the cost of having a controlled environment has become a major issue, and investigations have been conducted to develop cost-saving and efficient cultivation techniques. One research focus is on the utilization of stimulating appropriate photoreceptors for a certain time by far-red (FR) light to influence plant development. Here, we propose a novel laser biospeckle method, a non-destructive and real-time measurement method for the speedy evaluation of FR effects on arugula (*Eruca sativa* Mill) plants. Laser biospeckles are formed from the interference of scattered light from the organelles within the biological tissue, and the intensity of such speckles varies due to displacements within the tissue. In the experiment, while illuminating with FR and red (R) LED light of 735 nm and 630 nm, respectively, for a duration of 120 s to 300 s, the leaves of an arugula plant were irradiated simultaneously with laser light of 852 nm to obtain biospeckles. Video clips were recorded using a complementary metal-oxide-semiconductor (CMOS) camera at 15fps for 20 s. Correlations between the first and the subsequent frames were calculated to investigate the differences in the internal activity with exposure to FR and were characterized by a parameter named biospeckle activity (BA). Experiments were done with the healthiness and the age of the plant as parameters. It was found that depending on the healthiness of the plants, under short durations of 120s FR, BA and thus the internal activity within the leaves increased compared to the long duration of 300s FR. Further, a 1-month-old plant was found to have a faster decay of correlation and thus a steep increase in BA compared to that for a 3-month-old plant. Our results suggest that BA could be used as a measure to investigate the effects of FR or FR plus R in plant development within a timeframe of a few minutes, and thus can be employed as a complementary measurement technique for the speedier investigation of FR effects on plants.

## 1 Introduction

Global environmental changes, such as global warming, drought, and acid rain, are known to significantly impact plant growth and thus food yield (Prasad et al., 2002; Wong, 1990; Flannigan et al., 2000; Huang et al., 2022). With such climate change on the rise, sustainable agriculture is expected to become difficult in terms of plant resources and food security, which is a significant problem (Craufurd and Wheeler, 2009; Benke and Tomkins, 2017). In addition, the problem of the food supply is expected to accelerate further as the land available to grow plants is steadily decreasing due to reforestation, declining water supply, population growth, and climate change (Thomaier et al., 2015; Rajan et al., 2019; Hoenecke et al., 1992).

Vertical farms, where plants are grown in high-rise buildings with complete control of the surrounding environment, including light, temperature, and water, have attracted attention for their ability to significantly increase plant productivity. In such modern artificial agriculture, light is a key parameter, and control of parameters such as light intensity, duration, and wavelength composition is a challenge to increase the yield and value of products (Paradiso and Proietti, 2022 and references therein) (Demotes-Mainard et al., 2016; Tan et al., 2022). For instance, red light affects the photosynthetic apparatus development, and red and blue light are most efficiently utilized for photosynthesis (Hoenecke et al., 1992). Blue light influences stomatal opening, plant height, and chlorophyll biosynthesis, while far-red light (FR) stimulates flowering in long-day plants, and the red/far-red ratio regulates stem elongation and branching, leaf expansion, and reproduction (Jin et al., 2021). However, the cost of maintaining the environment is enormous, especially the cost related to lighting, which is a major obstacle to expanding the vertical farms (Jin et al., 2021; Zhen, 2020).

There existing research into improving the efficiency of plant growth through the use of fertilizers, improved lighting, and genetic improvement (Benke and Tomkins, 2017). Among these, irradiating plants with FR in the wavelength range of 700 to 800 nm has recently attracted increased attention to improve growth efficiency through the action of photoreceptors (Tan et al., 2022). However, it has been reported that the effect is higher when FR and red light are mixed than when FR alone is used due to the Emerson reaction (Emerson et al., 1957; Pettai et al., 2005).

Recently, it has been shown that in addition to the general photosynthetic wavelength range of 400~700 nm, irradiation with FR can increase growth efficiency. For example, Jin et al. reported that in lettuce cultivation, adding FR light to red and blue LEDs increased the dry leaf weight and leaf area of lettuce after 30 days of growth (Jin et al., 2021). Zhen (2020) also reported that adding FR to photosynthetic wavelengths (400~700 nm) during lettuce cultivation increased carbon gain and biomass by approximately 30% (Zhen, 2020). Kalaitzoglou et al. (2019) reported that adding FR treatments during tomato cultivation during the early growth stages increased productivity while mixing FR/R at a larger ratio than the natural light had a negative effect on growth.

The growth evaluation in the above cases is based on the measurement of plant growth in terms of physiological measures such as photosynthetic rate and physical measures, for example, plant height, leaf size, and weight of cut and dried leaves (Demotes-Mainard et al., 2016). However, it is a challenge to observe plants in a living state and also monitor short-time changes as the existing methods take considerable time with the need to wait for the plants to grow to a size that can be measured (Yadava, 2022; Lazzarin et al., 2024). In contrast, the laser biospeckle method is a technology that enables the non-destructive and non-contact evaluation of biological objects (Braga et al., 2009; Pandiselvam et al., 2020; Rajagopalan et al., 2021).

Minute irregularities comparable to the wavelength of the illuminating laser light in static objects cause light to scatter in different directions. When the scattered light reaches a screen or a camera, the scattered light then interferes, forming a random granular pattern of light-dark patches called speckles. When the laser light irradiates biological structures such a**s** a leaf, light gets scattered from the surface, including internal structures. Here, the internal structures refer to cellular structures such as the nucleus and mitochondria that are involved in the functioning, and the sizes are in the order of wavelength of light or much smaller. Such organelles scatter light and the scattered light interferes with producing speckles on the observation plane, for example, on the camera plane. Because the structures within the tissue are changing, the speckle pattern also changes in relation to the movement within the leaf or seed itself. Such speckles obtained from a living organism are called biospeckles (Aizu and Asakura, 1996).

Here, biospeckles are considered to be related to cellular activities resulting in changes in the shape of the organelles themselves, for example, cell expansion or related to movement such as nutrient transport (Zdunek et al., 2014; Silva et al., 2018). The intensity of the biospeckle changes with the movement of organelles within the tissue. These changes are proportional to the movements with larger change in response to more significant movements. By analyzing the intensity changes of the speckles, it is possible to infer changes within the plant tissue. It should be pointed out that although the biospeckles change with the changes in biological activity due to environmental factors, the exact origin of biospeckles remains unknown.

Biospeckles have been used to evaluate the quality of fruits and vegetables with a few root-related studies in the agricultural field (Zdunek and Cybulska, 2011; Zdunek et al., 2014; Singh et al., 2020; Costa et al., 2017). Our group has recently demonstrated the application of laser speckles in the investigation of plants. Hirai et al. demonstrated that the response of plants to different sound frequencies could be rapidly evaluated by laser biospeckles (Hirai et al., 2020; Rajagopalan et al., 2021), indicating the potential of laser biospeckles in speedy monitoring in plant studies.

The objective of the current study is to investigate the applicability of the laser biospeckle method in the speedy evaluation of the short-term effects of far-red light on arugula plants [an extension of a report in a conference (Igarashi et al., 2023). To demonstrate the feasibility of the proposed method, we have used the healthiness and the age of the plants as parameters and conducted an investigation on short exposure of FR for 2 or 5 minutes. Compared with the existing techniques of measurement by CO2 assimilation, the proposed method is non-invasive and could be complementary to the existing techniques.

## 2 Materials and methods

### 2.1 Plant sample

Arugula or rocket plant (*Eruca sativa* Mill), which grows quickly, is easy to cultivate, and can be grown in a variety of environments, was used as the plant sample in the experiments. A 3 cm square cube of hydroponic rockwool (0138-008, Grotop Grodan) was cut crosswise, and 3 to 4 seeds were sowed. The rockwool was placed in a 100 ml disposable cup with three holes in the bottom to absorb water, and approximately 10 plants were grouped in a tray and filled with enough water for the rockwool to absorb. The seeds were soaked in 3% oxindole for 10 minutes immediately before planting and then rinsed twice with water.

A custom-made cultivation chamber equipped with a temperature and humidity controller (APISTE, PAU-300S) a fluorescent lamp (SMILE, XFX2-20W), and a plant lamp [HUJIKURA plant light (HUJIKURA, KY-08W-SC) at a temperature of 23 ± 5˚C and a humidity of 70 ± 5% was used for growing the plants. A day-night cycle of 12 h/12 h with photosynthetic photon flux density (PPFD) levels of 186 μmol/m^2^s for the day period was used. The nutrient solution was given once a week, and it was made with 1.5 g of fertilizer (OAT House No. 1) and 1.0 g of manure (OAT House No. 2) dissolved in approximately 20 L of water to have a conductivity of 1.0 ds/m as measured by a water quality meter (FUSO Corporation, Model-7200).

**Figure 1:**
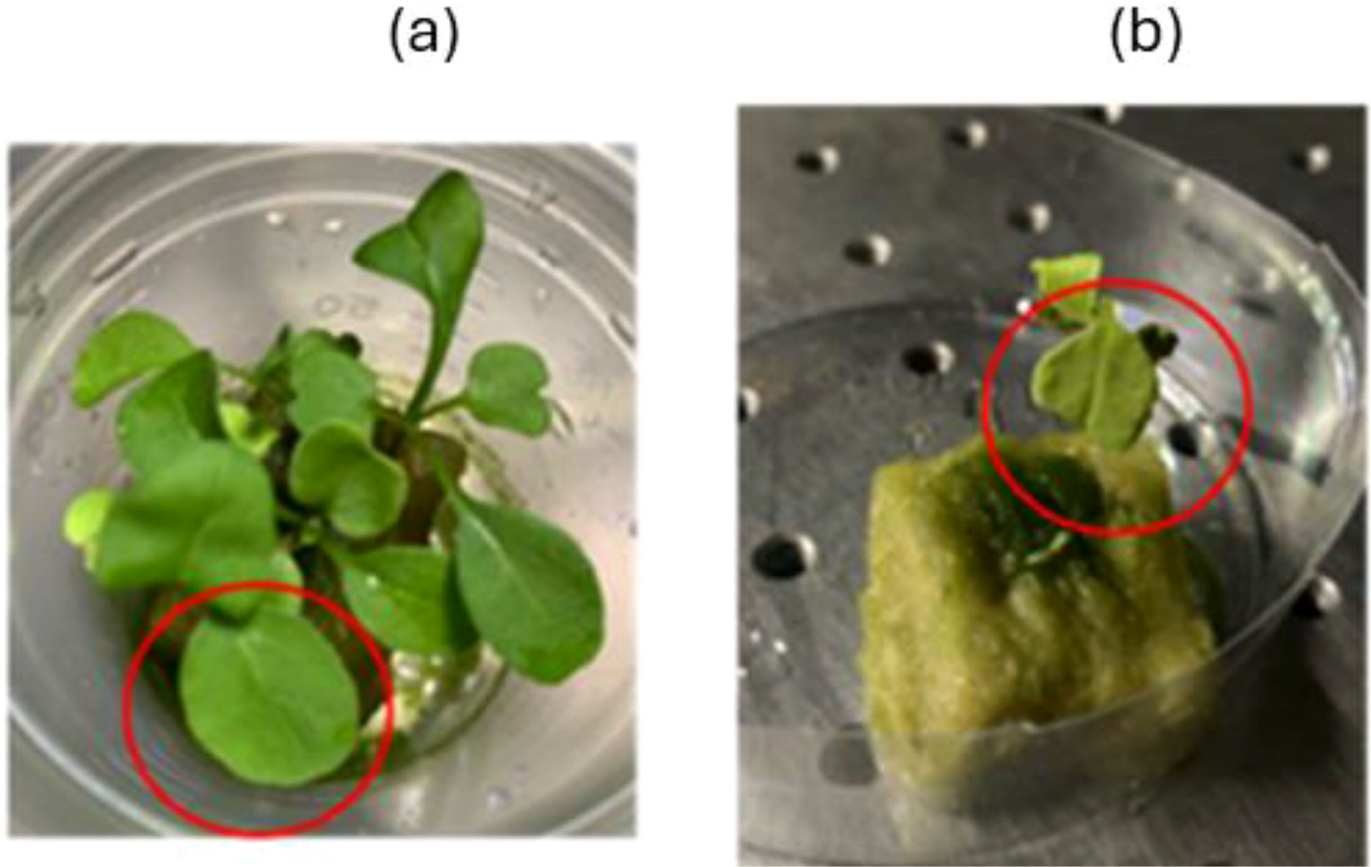
Argula samples of different health conditions with **(A)** healthy and **(B)** weak individuals. Red circles indicate the measured leaf region. Both were grown in the custom-made plant chamber in the laboratory under controlled conditions.

**Figure 2:**
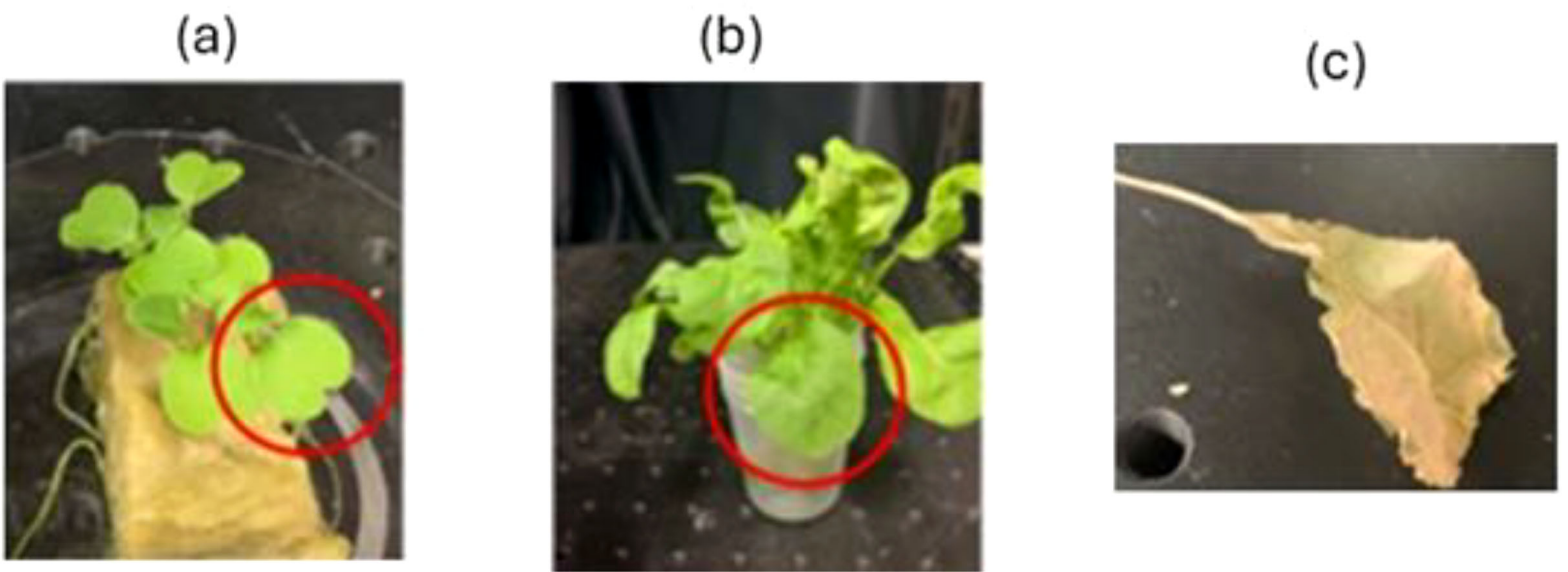
Argula samples used to study age effects with pictures of seedlings at **(A)** 1 month old and **(B)** 3 months old and **(C)** a dying leaf turning yellow that was detached from the plant just before the experiment. Red circles indicate the measured leaf region.

We conducted two sets of experiments, one investigating healthiness with different health conditions and the other investigating the effects of age with two different age groups. Representative examples used in both the experiments are shown in **Figures 1** and **2**. We selected (a) healthy individuals with green leaves and (b) weak individuals with yellow leaves from arugula grown from seed in the laboratory for approximately 1 month as samples. Hereafter, (a) and (b) will be referred to as healthy and weak, respectively. **Figure 2** shows the different-aged plants with a, b, and c representing, respectively, a 1-month-old, a 3-month-old, and a dying plant. Here, a leaf that had turned yellow and detached from the plant was referred to as a dying leaf. For both experiments, three replicates were prepared for each of the sample conditions.

### 2.2 Experimental system and protocol

**Figure 3:**
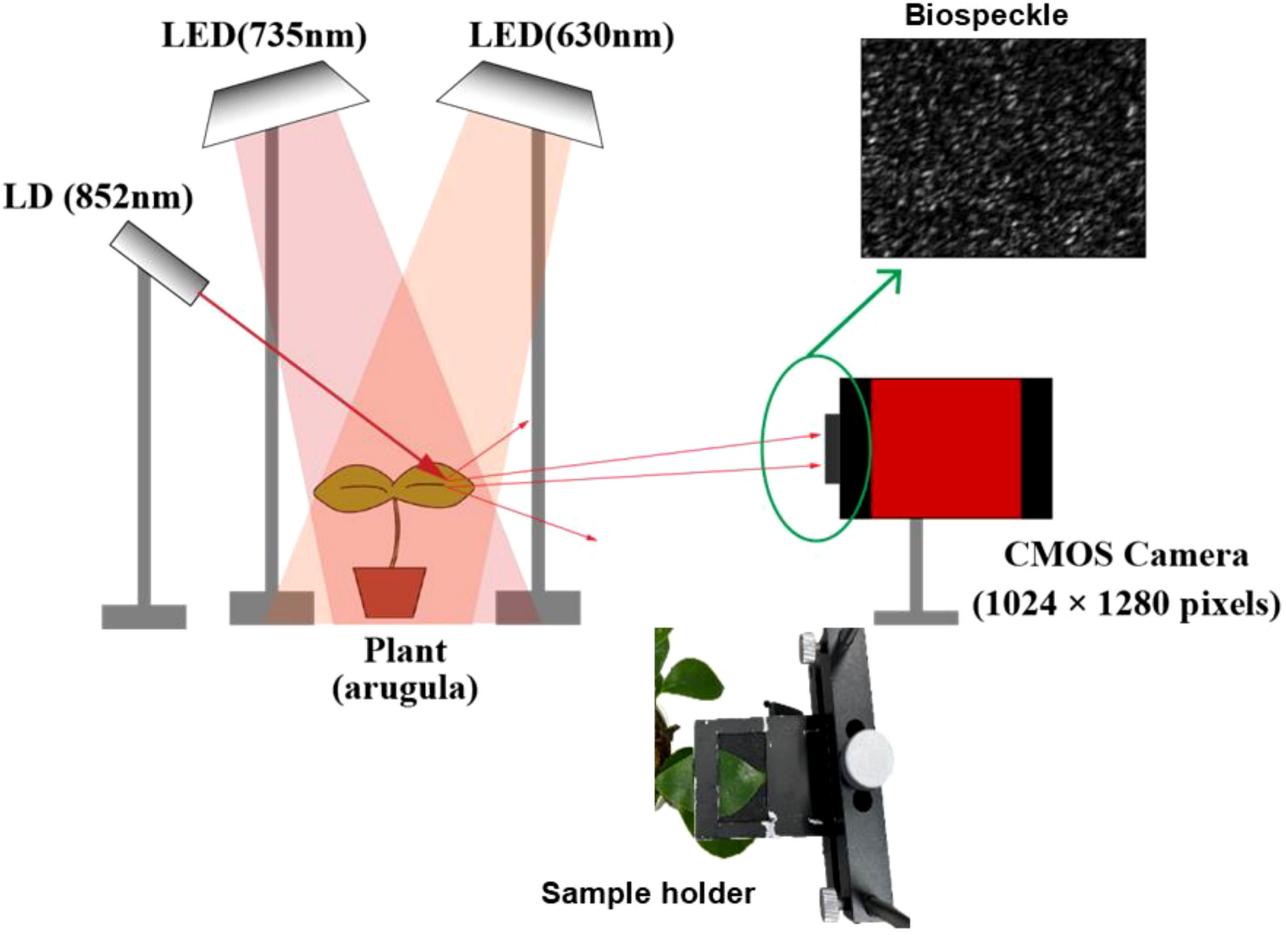
Experimental system for measuring the short-term FR response of plants using the laser biospeckle method with an inset of the sample holder with a mounted leaf. Laser light of 852 nm was made to illuminate the leaf of the plant, and the scattered light from the leaf was acquired by a CMOS camera. The plant was illuminated either by FR (735 nm) alone when investigating the healthiness effect or FR plus R (630 nm) illumination in the age effect experiment. The biospeckles shown in the inset formed on the camera plane were recorded as videos to be analyzed.

**Figure 3** shows an overview of the experimental system. The system contained two light systems, one for illuminating the plant sample and the other for measurement by laser speckle. The illuminating system consisted of an FR LED light with a wavelength of 735 nm fixed with an arm stand that irradiated a leaf of the sample plant. A red LED of 630nm was also used for experiments done for the FR plus R condition. The system for measuring speckles consisted of a laser diode (LD) (THORLABS, CPS series, NewJersey, USA) with a wavelength of 852 nm, a beam diameter of 1 mm × 2 mm, and a power of 1.26 mW. The plant leaf was gently held by sandwiching the sides of the leaf to magnetic sheets, as shown in the inset of **Figure 3**. The scattered light from the leaf interfered to produce speckles on the surface of the CMOS camera (THORLAB, DCC1545M-GL, NewJersey, USA; pixels: 1024 × 1280). The distances between the laser and the sample and between the sample and the CMOS camera were each 150 mm.

Laser light was made to an incident on the leaf at 45 degrees with respect to the normal of the leaf, and the scattered light was collected by the CMOS camera. Here, the basic configuration of the optical system was adjusted so that the light reflected from the sample leaf interfered. As for the distances, the distance from the leaf has to be far enough that the scattered field interfering with the camera plane produces fully-developed speckles (Dainty, 1984). A sharp-cut filter (IR82, cutoff wavelength: 820 nm or less, Fujifilm, Japan) was placed between the plant sample and the camera to block unwanted light other than the scattered LD light from the sample entering the camera.

**Figure 4:**
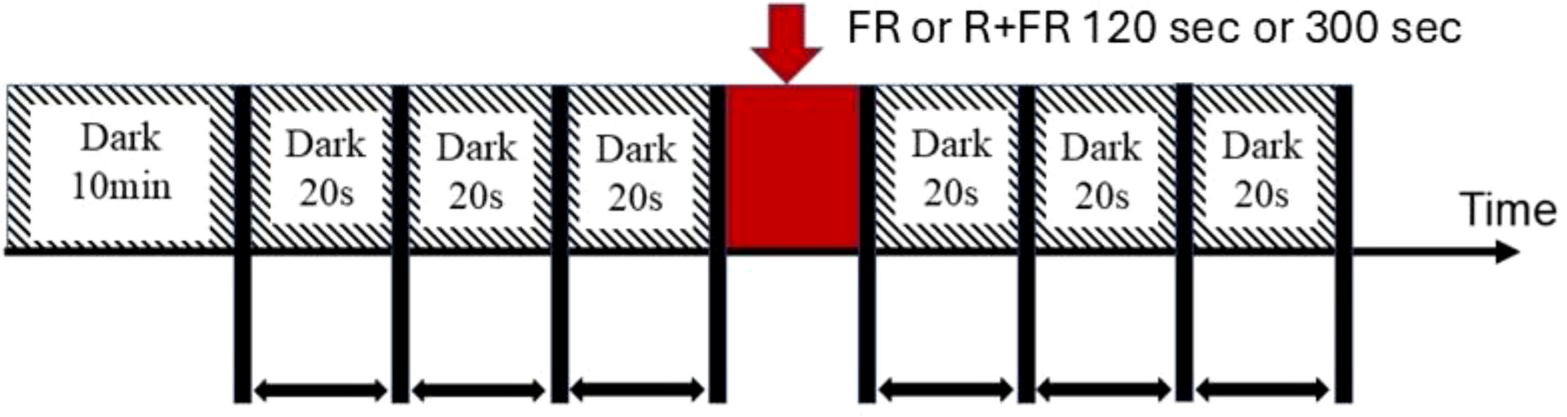
Experimental protocols for comparing biospeckles at different FR or FR plus R irradiation. Following an initial dark period of 10 min, the biospeckles were recorded. Similarly, following FR or FR plus R radiation for a period of 120 s or 300s, the biospeckles were recorded. In both cases, the recording was done in sets of three with each set lasting for 20s and thus for a total of 60s.

**Figure 4** shows the experimental protocol used to acquire the images. The CMOS camera recorded speckle video clips at a frame rate of 15 fps. Before the start of the experiment, the plant was taken out of the chamber and kept in the dark for 10 min, followed by the acquisition of biospeckle videos. This was followed by exposure to FR. The FR exposure experiment consisted of two investigations. The first investigated the healthiness and FR effects and the second age effects under FR plus R. In both cases, the FR or the FR plus R exposure periods were varied and done at an exposure period of 120 s or 300 s.

In both experimental cases, biospeckle acquisition was done in a consecutive sequence of three sets, with each set lasting for 20 s, totaling 60 s. FR and R light PFD levels were set at 78 µmol m^−2^ s^−1^ and 186 µmol m^−2^ s^−1^, respectively. All the light levels used during the growth and FR exposure conditions are given in **Table 1**. Here, the FR and R PFD light levels were chosen based on the previous research of Jin et al. (2021). Furthermore, the timing of 120 seconds and 300 seconds for the laser speckle study was chosen based on our previous laser speckle response of plants to sound study (Hirai et al., 2020) and the short-term effects investigated by Lazzarin et al. (2024). The conditions for the experiments are summarized in **Table 1**.

**Table 1:** Illumination conditions under different conditions of laser biospeckle recording in experiments with healthiness and age parameters and the summary of results.

Experimental parameters	FR/R PFD (μmol m^-2^s^-1^)	Sample details	Exposure time (s)	BA slope results at 2 sec
Healthiness of the plant	78/0	Healthy	120	Increase
			300	Decrease
		Weak	120	Decrease
			300	Decrease
Age of the plant	78/186	1 month old	120	Increase
		3 months old	120	Decrease

### 2.3 Laser biospeckles and analysis

As explained in the introduction, biospeckles are formed when living tissue, such as plant leaves, is irradiated with laser light. This is because the light scattered from the surface and from the internal structures of the leaves interfere to produce speckles on the camera surface. Several methods exist for analyzing biospeckles, including a contrast-based evaluation of each acquired speckle image and assessment of the uniformity of speckle images using the second-order moment (IM) (Alves Braga Junior et al., 2007). In this study, we conducted cross-correlation between the frames of the acquired biospeckles (Ansari and Nirala, 2013) to evaluate the response of the plants to light.

The frame rate of the camera during the measurement was 15 fps, and video data was acquired in three sets, with each set lasting 20s for an overall period of 60 s. The video data were analyzed using analysis software (MATHWORKS, Natick, MA, USA version: R2023b). Here, each 20-s video was analyzed individually (**Supplementary Information S1**, **S2**), and the first 20-s video was used for comparison between different conditions, namely the healthiness and the age of the plants. **Supplementary Videos** for the dead leaf and 1-month-old leaves under no FR and FR are given in S3, S4, and S5, respectively.

In the analysis, the correlation coefficient denoted by *γ* was calculated between the first frame, which is reference frame A, and the subsequent frame, B. A_mn_ and B_mn_ are the intensities, where m and n specify the pixels in the frame. Ā and $\bar{B}$ indicate the average of the intensities of each of the frames *A* and *B*, respectively.


(1) $$\begin{array}{c} \gamma =\frac{\sum_{m}\sum_{n}\left(A_{mn}-\bar{A}\right)\left(B_{mn}-\bar{B}\right)}{\sqrt{\left\{\sum_{m}\sum_{n}{\left(A_{mn}-\bar{A}\right)}^{2}\right\}}\left\{\sum_{m}\sum_{n}{\left(B_{mn}-\bar{B}\right)}^{2}\right\}} \end{array}$$

When there is greater movement within the leaf, there is higher decorrelation and *vice versa*. Thus, a decrease in correlation and an increase in correlation relate to large and small changes, respectively. In other words, a decrease in correlation corresponds to increased plant movement and an increase in correlation corresponds to decreased plant movement. Therefore, to create a positive measure that can relate to the activity of the plant, we used a quantity, the biospeckle activity (BA), which is calculated as the value that remains after the correlation coefficient is subtracted from unity. BA makes it easier to see changes over time.


(2) $$\begin{array}{c} BA=1-\gamma \end{array}$$

For stationary objects, BA is zero, while for an object that moves, BA decreases with the slope of variation depending on the motion of the object. When the object is moving fast, the slope is steep, and when the object moves relatively slow, then the slope of BA is gentle. For comparison with the plant leaf, a paper object, as a stationary object, has been introduced to assess the relative activity between the reference or the first frame and the rest of the recorded video frames.

To quantitatively evaluate the slope of the BA variation, the variation within the first 0 to 2 s was used, and the slope over this region was calculated by fitting a straight line. The fitted slope was used as a parameter and is indicated as a shaded area in all the graphs. Based on the slope, we can discern when the initial slope of BA variation is steeper, the speckle frames are then decorrelated faster, and thus, the activity or movement of the organelles within the leaf is faster. In contrast, when the slope of the initial variation is gentler, the neighbouring frames are then correlated, and thus, there is slower movement or there could be more coordinated displacements of the organelles within the leaf. When there is zero movement between the frames, then the correlation becomes close to one and the object could be considered as stationary.

### 2.4 Statistical analysis

Statistical analysis was done using Excel (Microsoft, Seattle, DC, USA). A paired t-test was performed to assess whether there was a significant difference, set at p< 0.05. For each of the conditions, BA was averaged across three samples. Statistical significance was tested between the treatment conditions of before FR or FR+R and after FR or FR+R treatments. In the healthiness effects experiment, significance tests were conducted between healthy and non-healthy plants. Similarly, for the age effects experiment, significance tests were conducted between 1-month-old and 3-month-old healthy plants under no FR and FR+R.

## 3 Results

### 3.1 Healthiness of plant and FR

**Figure 5:**
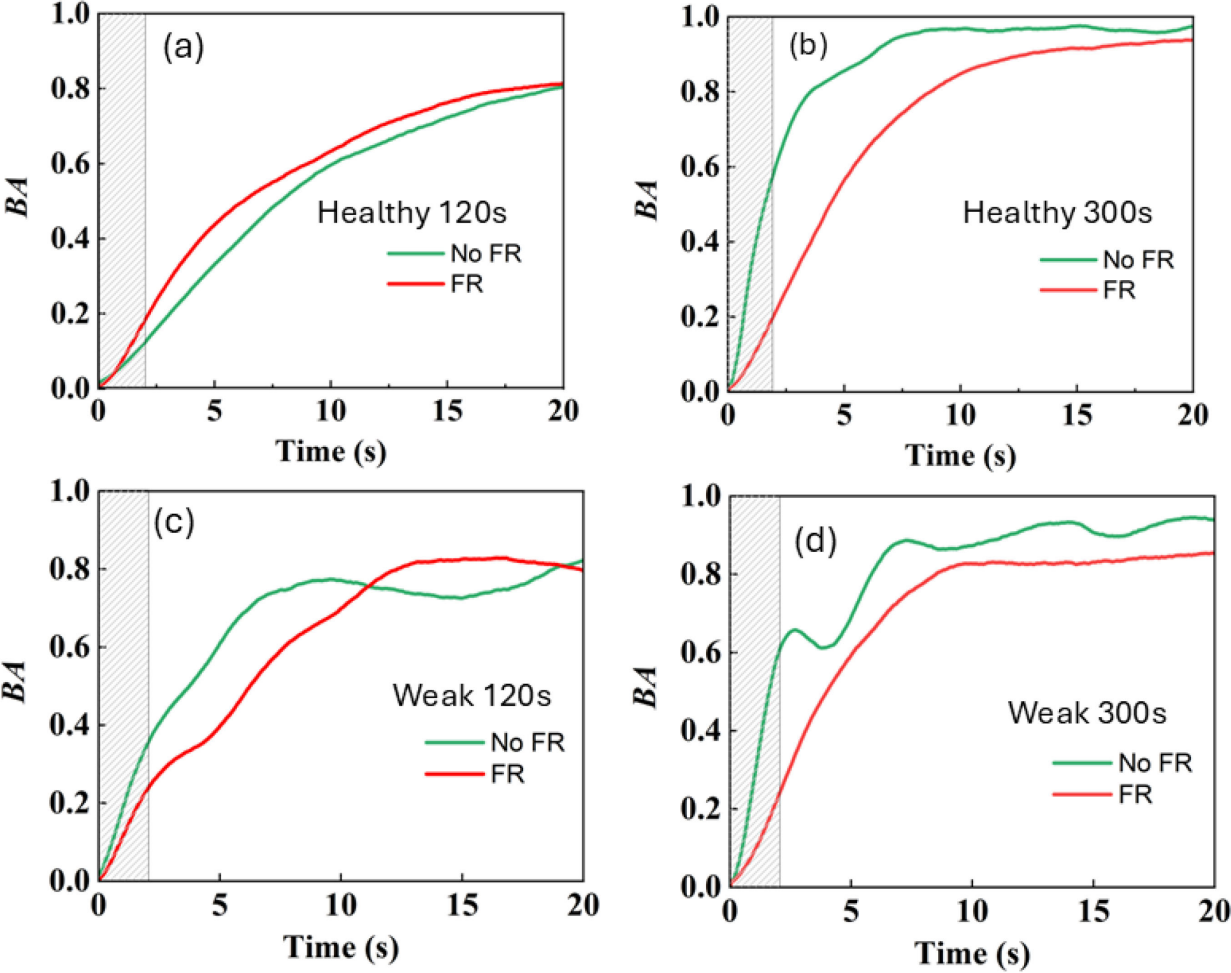
Average BA as a function of recording time for healthy **(A, B)** and weak **(C, D)** plants at different FR irradiation times of 120 s **(A, C)** and 300s **(B, D)**. Green and red lines indicate NoFR and FR exposure conditions. The shaded area corresponds to the region used for slope evaluation.

The results of the BA obtained for the healthy and weak plants that were identified from the leaves as indicated in **Figure 1** are shown in **Figures 5A–D**, respectively, under FR-only irradiation of 120 s (a,c) and 300 s (b,d). In all the plants, the BA increased with the increase in time. However, the slope of the rise of BA was different depending on the healthiness of the sample and the FR exposure times. When comparing the 120 s and 300 s FR exposures, there was a clear difference in the increase of BA depending on the healthiness of the sample.

Under 120s FR irradiation, the BA variation before and after FR exposure differed for healthy and weak plants (**Figures 5A, C**). For the healthy sample, the slope of the variation of BA was steeper after exposure than before, indicating a stronger activity within the healthy leaf (**Figure 5A**). In comparison, for the weak sample, the slope of the variation of BA was steeper before than after exposure to FR (**Figure 5C**). This implies that FR exposure could be causing less activity within the leaf of a weaker plant or slowing it down, possibly because of less biological activity within the leaf.

In contrast, under 300 s exposure to FR radiation, BA variation before and after exposure was similar for both the healthy and weak samples, with the slope being lesser for the FR-exposed ones (**Figures 5B, D**). In other words, the BA before exposure was steeper than that after exposure. Moreover, the variation in slope was also statistically significantly different depending on the healthiness of the plant. For the healthy plant, there was a significant difference in the slope between the NoFR and FR exposed samples. These results indicate that the effect of FR was different depending on the health status of the plant.

#### 3.1.1 Results obtained with paper as a control

**Figure 6:**
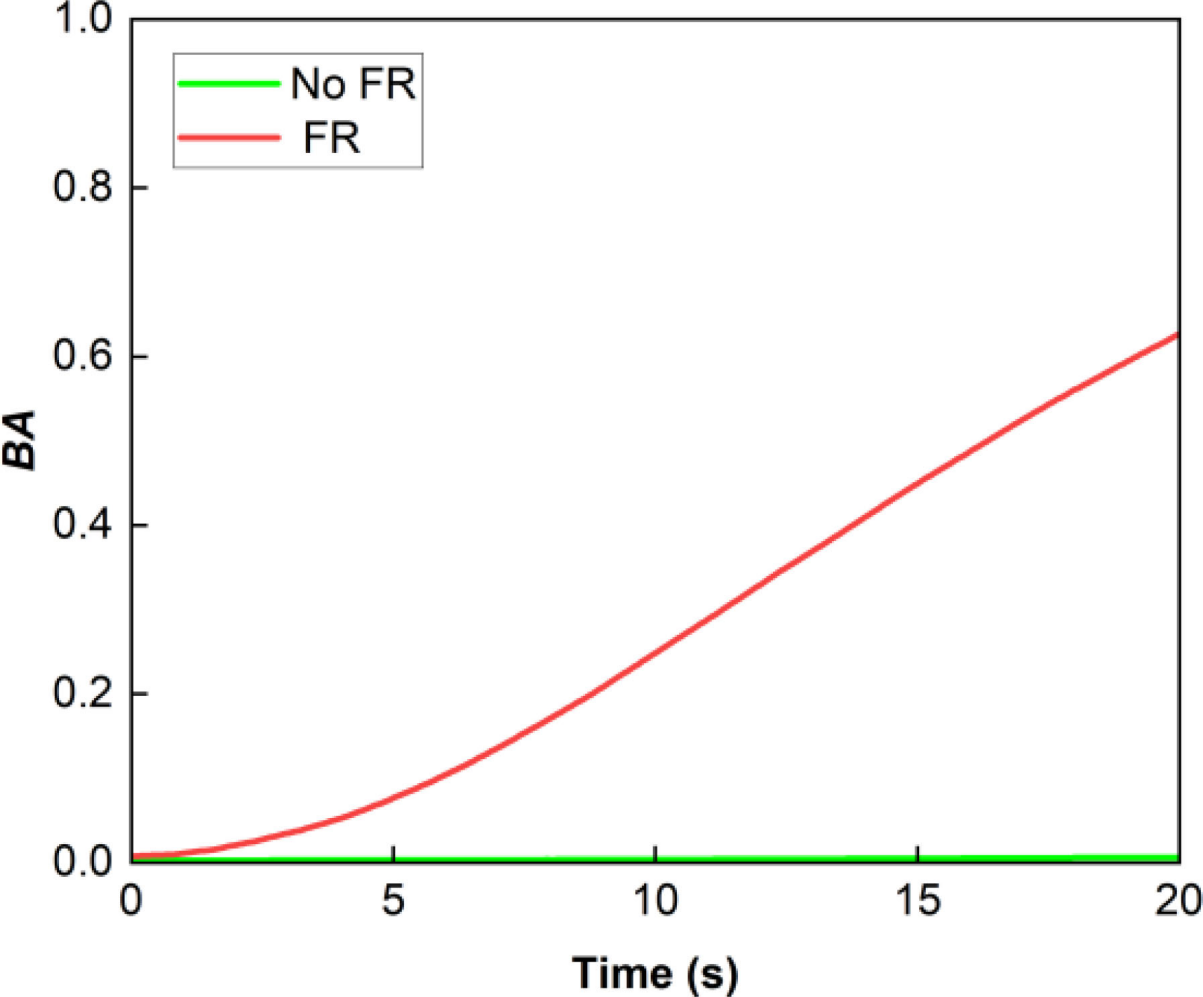
Average BA as a function of time for paper. Although we expected the paper to show null activity because of the absorption of FR light by paper, the activity within the paper was not zero, but there was a finite correlation between the recorded frames. Under no FR, the correlation was one all the time or, in other words, BA was zero.

For comparison with the plant leaf, a stationary paper object was introduced to assess the relative activity between the reference or the first frame and the rest of the recorded video frames. **Figure 6** shows the results obtained under the absence and presence of FR. Under no FR, as shown by the green line, BA was zero or, in other words, the correlation was one all the time as expected. Under FR, although we expected the paper to show null activity because of the absorption of FR light by paper, the activity for the paper was not zero. There was a finite correlation between the recorded frames and there was a finite BA that increased slowly with time. This was believed to be due to the heating up of the paper due to heat from the FR radiation.

### 3.2 Evaluation based on the slope of BA variation

Next, the slopes of BA variation in **Figures 5A–D** were quantitatively compared. To evaluate the difference in the steepness of the variation of BA under different conditions, the slope of the BA variation was estimated as defined in the analysis section and was used. As stated previously, the steeper the variation, the faster the correlation between successive frames vanishes, and thus, the motion could be more extensive, and the activity could be stronger. In contrast, the slower variation or lower value of the slope could be the result of a slowdown of the activity and could be from sustained correlated activity. It thus could be the result of displacement activity within the plant tissues, such as expansion and elongation growth.

In fact, a plant consists of thousands of cells of different shapes, sizes, and so on, and the growth is uniform across the plant. At the same time, plant growth is a complex and highly dynamic process and is stringently regulated with proper balance between turgor and wall extensibility. Turgor pressure makes the cell swell. Turgor occurs because the osmotic water uptake causes the cells to become turgid, leading to internal pressure. Thus, it is because of the turgor pressure that a cell grows or elongates. The turgor-driven cell wall expansion is irreversible and involves a slow reorganization of the cell wall (Hamant and Traas, 2010); (Taiz and Zeiger, 2003).

**Figure 7:**
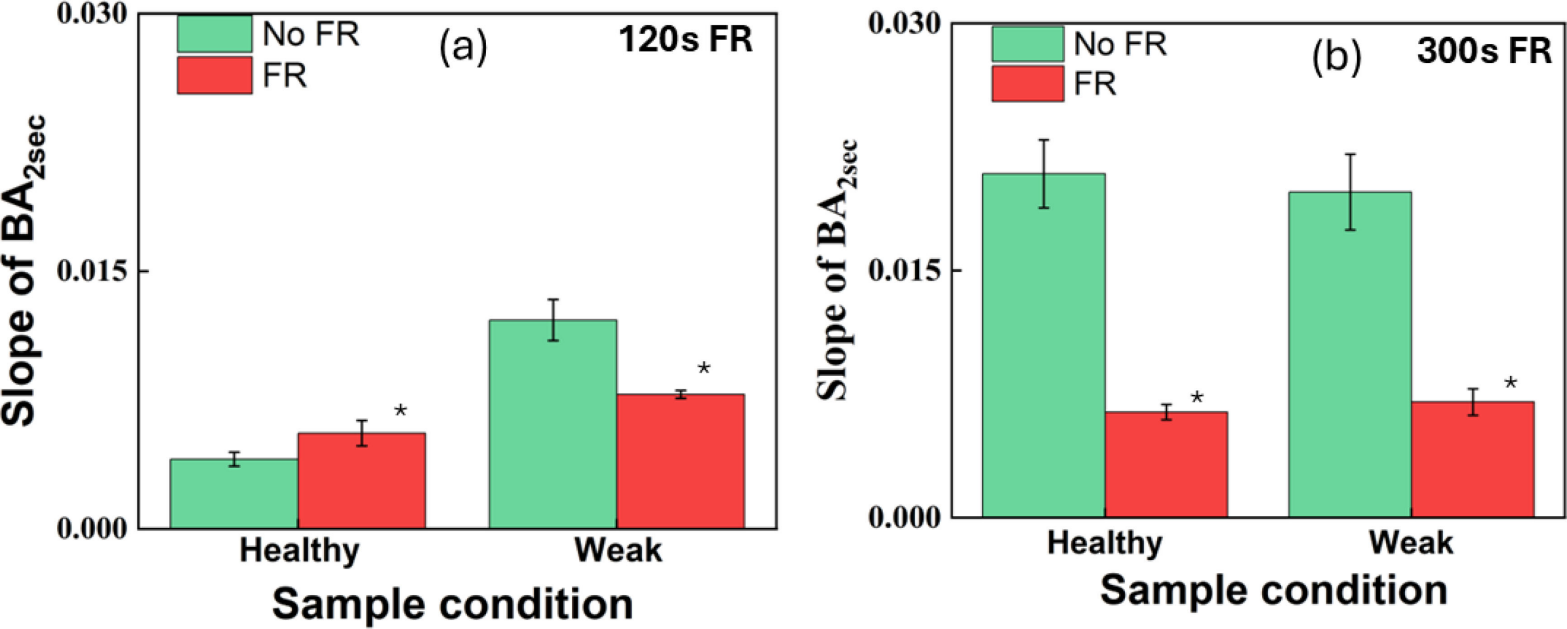
Comparison of the initial slope of average BA up to 2 s at different FR irradiation times shown in red for **(A)** 120s and **(B)** 300s compared to no FR irradiation shown in green. Error bars correspond to standard error. Here, the slope was calculated from the initial 2s by fitting a line to the region within the shaded area of **Figure 5**. * indicates a significant difference between the NoFR (green bar) and FR exposure conditions (red bar) for both **(A, B)**. Further, we expected the slopes of the healthy and weak plants under no FR to behave in the same fashion. However, the weak set and the healthy sets were prepared separately; it is possible that there existed large differences.

The slope under different FR exposure times, i.e., 120 s and 300 s, is shown in the histogram plots in **Figure 7**. The vertical axis represents the slope within a period of 2s, and the horizontal axis represents the sample condition, with the green and red bars indicating the before and after FR conditions, respectively. For 120 s of FR irradiation, for the healthy young plant, the slope of BA following FR exposure was large in comparison to that in the absence of FR. In contrast, for the weak sample, it is *vice versa* with a larger slope under no FR than that of under FR exposure. A paired t-test revealed that there was a significant difference between the healthy and weak plants and between the exposure conditions for 120 s. For the 300s experiment, there was a significant difference between the exposure conditions but not between the weak and healthy conditions.

For the 300 s FR exposure, irrespective of the healthiness of the leaves, the slope of BA was smaller under FR than that under the absence of FR. The steepness of the slope indicates the activity within the tissue, and the results indicated that longer FR exposure slows down the activity. When comparing 120 s and 300 s FR exposure times, longer FR exposure led to a decrease in the slope irrespective of the healthiness of the plant. For both healthy and weak samples, the decrease was almost two-fold. One possibility was that, with longer exposure, there could be an effect of heat generated from longer FR exposure, which may slow down the internal activity within the leaf tissue and possibly influence growth.

### 3.3 Plant age and FR plus R

**Figure 8:**
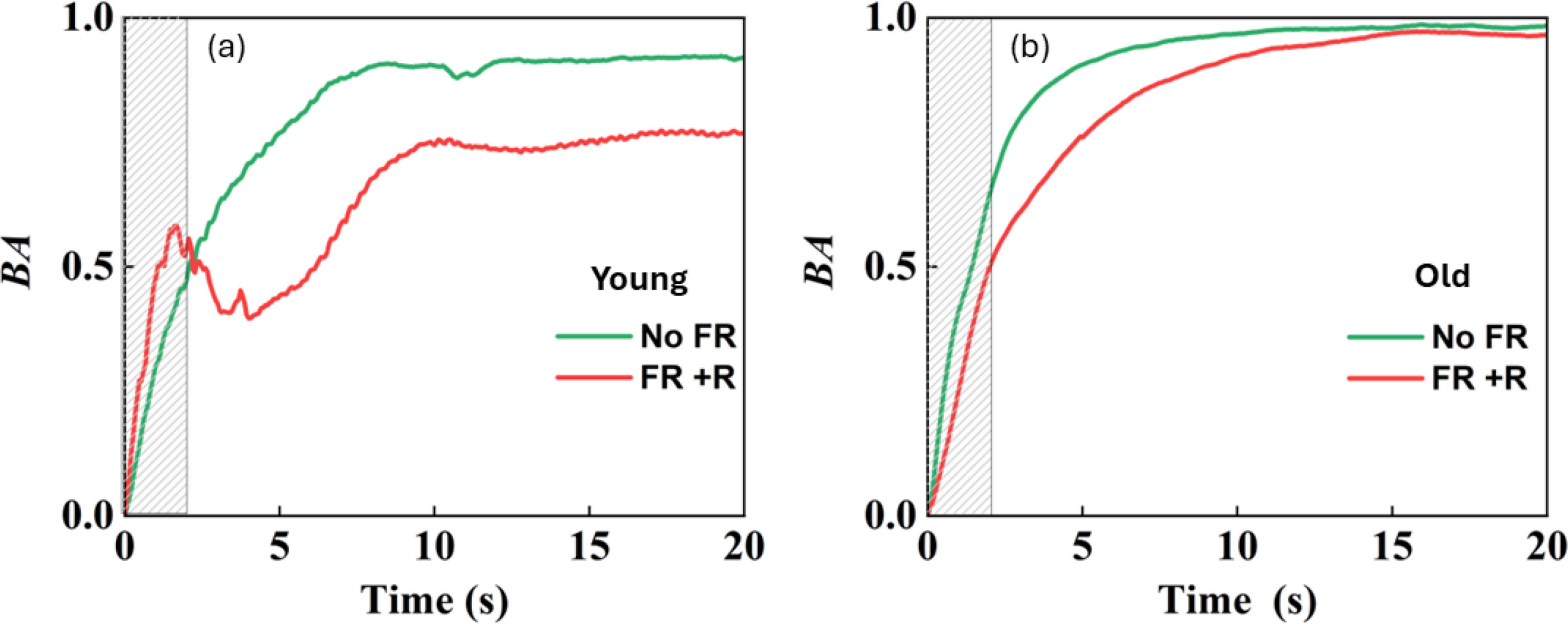
Average BA results under no FR and FR plus red light for 1-month-old **(A)** and 3-month-old plants **(B)**. Paired t-tests were done between 1-month-old and 3-month-old plants under NoFR and FR+R and the results differed significantly.

**Figure 9:**
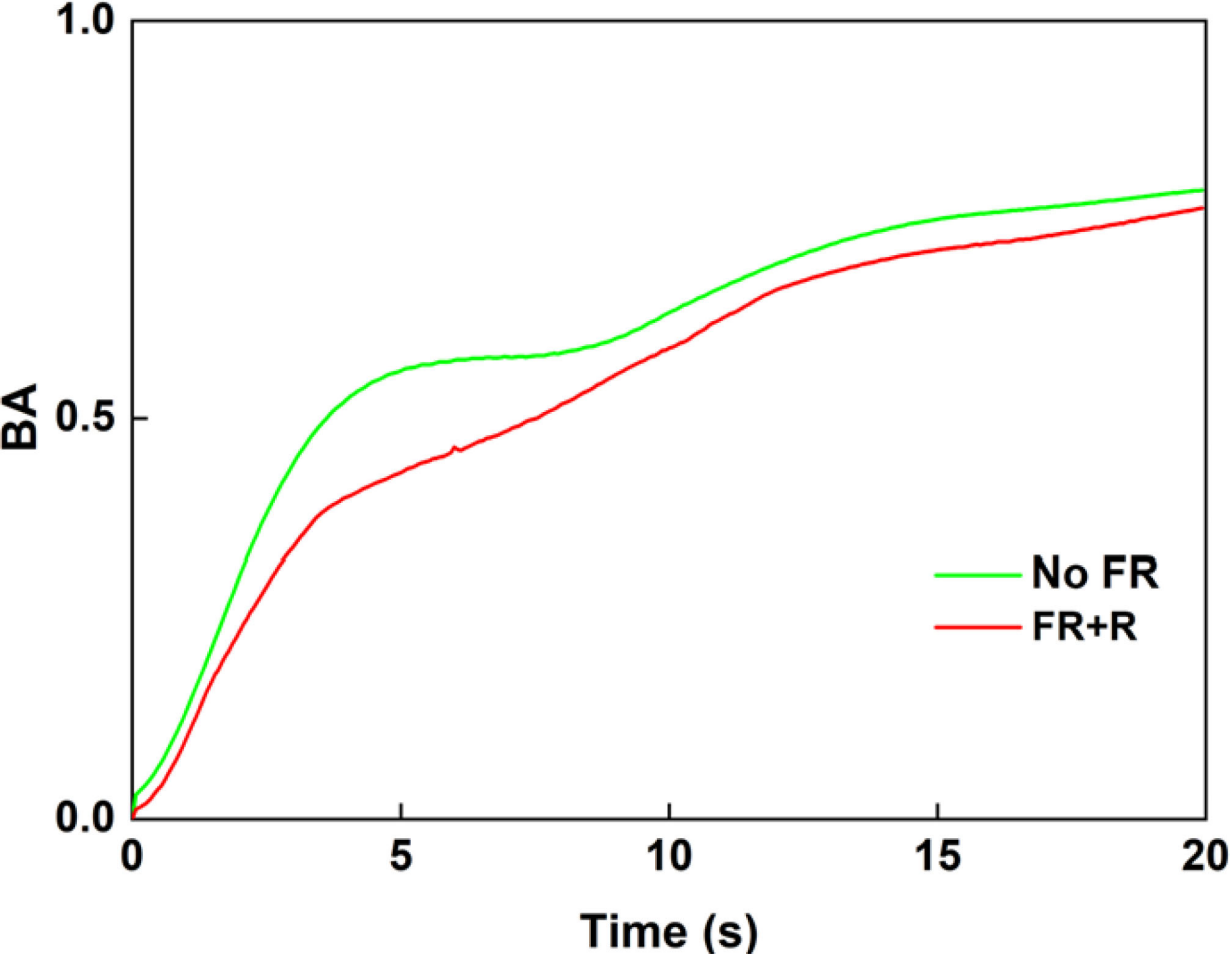
BA as a function of time in seconds for a dying leaf under NoFR and FR plus R. Despite the leaf having fallen off, there was some difference in the BA activity depending on the exposure to FR+R compared to no FR. The dying leaf experiment was done to confirm the aging effect.

The influence of FR plus R exposure on the age of the plant was also investigated. **Figures 8A, B** show the BA results obtained under the presence and absence of FR plus R, respectively, for 1-month-old and 3-month-old plants. The vertical axis represents BA and the horizontal axis represents the measurement time. FR plus R exposure was given for 120 s. For comparison, the dying plant leaf is also shown in **Figure 9**, and it can be seen that the activity of the dying leaf was much slower. Here, the dying leaf is the one that had fallen off and is no longer attached to the plant, and the appearance was also yellow. Thus, a clear correlation between the age of the plant and FR plus R effects can be seen from a comparison of the BA variation. For a plant of a younger age, the slope was steep with exposure to FR plus R. In contrast, for a plant of old age, the slope and the variation in BA are gentler, indicating the slowing effect of FR plus R for older plants. These results indicate that there is a clear effect of age on the FR plus R exposure.

**Figure 10:**
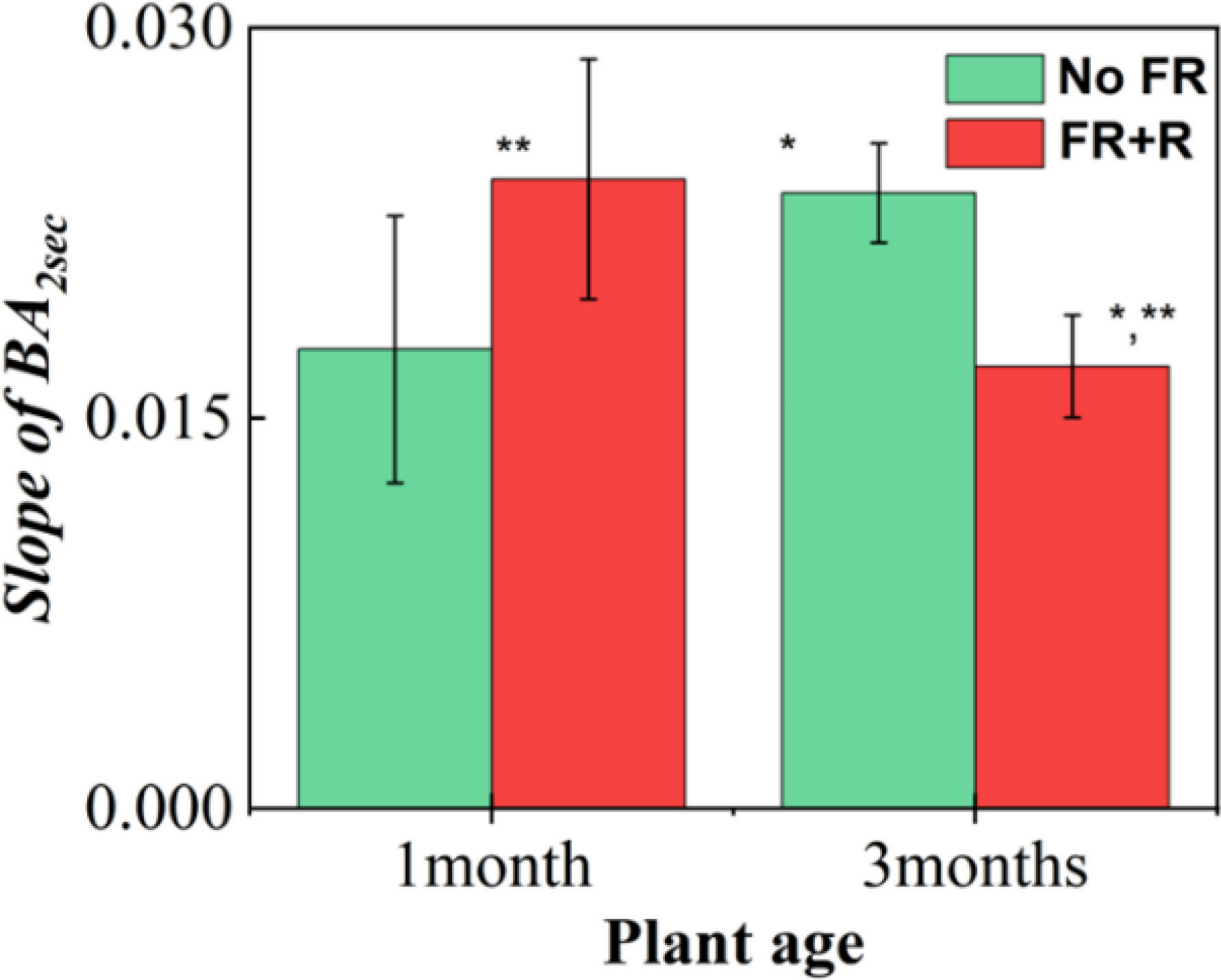
Slope of BA up to 2 s for two different plant ages, namely 1-month-old and 3-month-old plants. Green and red indicate the conditions of NoFR and FR+R exposure. In this experiment, FR was irradiated in conjunction with red LED light. Error bars correspond to the standard errors. * indicates the existence of a significance difference in the slope of BA between the ages of the plant. ** corresponds to a significant difference between the NoFR and FR+R treatments. Here, the slope was calculated from the initial 2s as shown by fitting a line to the region within the shaded area of **Figure 8**.

The results for the initial slope within the first 2 s for two different ages are shown in **Figure 10**. In the case of young leaves from a 1-month-old plant, the FR plus R treatment increased the slope while the slope decreased for the 3-month-old plant. This indicates that FR plus R exposure for a younger plant had the effect of increasing activity within the tissue. In contrast, for the 3-month-old plant, FR plus R had the effect of reducing the slope with increased age, indicating that there was a slowing down of activity within the plant. Significance tests using paired t-tests revealed that the slopes between the exposure conditions for both age groups were significantly different. * indicates a significant difference between age groups and ** indicates a significant difference between the NoFR and FR+R exposure conditions. This may be due to the fact that the younger leaves (1 month old) were more vigorous and responded more actively to the FR plus R stimulation. The other possibility is that, with age, the plants themselves stop making use of the FR and, thus, there was a reduction in the relative activity (review work of Tan et al., 2022).

## 4 Discussion

In this study, our aim was to investigate the short-term effects of far-red light on plants using the laser biospeckle method. In comparison to the existing techniques of physiological measurements by photosynthesis and physical measurements, we have shown that our non-invasive and non-contact laser biospeckle method can detect the variation response in the plant after a few minutes of exposure to FR and red light, and it can work as a complementary technique to the existing techniques. To our knowledge, the application of laser biospeckles to investigate instantaneous spatially localized changes after FR or FR plus R exposure has never been done before.

In our study, exposure of arugula to FR (735 nm) and FR plus R (630 nm) against NoFR were compared. To investigate the effects of FR plus R using laser biospeckles, a near IR laser light from a laser diode of 852 nm was used to obtain a video of biospeckles for a period of 20 s from the leaf of the arugula plant *in vivo*. Laser biospeckles are generated when scattered light from biological tissues interferes, and the intensities of such speckles change over time. Investigating the temporal correlation of speckles reflects the structural changes of the scattered structures within the biological tissue. When there is considerable variation, there will be less correlation and *vice versa*. In other words, by examining the decorrelation time of the time-varying speckles for a period of 2 s, we could differentiate the effects of healthiness and age when exposed to FR. The results obtained are summarized in **Table 1**, along with the conditions of the experiments.

We found that for healthy plants, there was a faster decay of the speckle correlation or steep increase in BA in comparison with an unhealthy plant of almost the same age (**Figure 5**). By investigating the slope within a 2 s window, under 120 s FR exposure, we found that FR has the effect of increasing the slope for a healthy plant (**Figure 7A**) but it decreases for a weak plant. Under longer FR exposure, there was a decrease in slope for both the healthy and weak plants (**Figure 7B**). As for the age comparison, a 1-month-old plant was found to have a faster decrease in correlation, and thus a steep increase in BA in comparison to that of a 3-month-old plant (**Figure 8**). This indicates that with increased age, there is a slowing down of activity within the plant (**Figure 10**). Thus, based on our results, we demonstrated that our method has significant sensitivity differentiating the response of the plant within minutes after exposure to FR or FR plus R. This is in agreement with our previous studies of plant’s response to sound where we could show that the laser speckles are sensitive to a minutes exposure to sounds (Rajagopalan et al., 2021).

Although this study was done with the intention of demonstrating the feasibility of the laser biospeckle method in the evaluation of FR/R illumination effects, it has the potential to extend to different species. FR is known to be species-dependent (Kalaitzoglou et al., 2019; Shibuya et al., 2016; Lazzarin et al., 2024) and also age plays a vital role in the response of a plant to FR. Thus, exposure times of FR are vital for the advantageous use of FR. One more advantage of our method is that it can be applied to investigate the different parts of a plant. The current study was restricted to leaves. Our method allows us to study the effects of localized changes in relation to perception sites and inter-organ relationships and might be useful for applications in horticulture, such as applying supplemental light at precise times and locations, helping to optimize energy use (Demotes-Mainard et al., 2016).

## 5 Summary

We proposed using laser biospeckles to investigate the short-term effects of FR. The laser biospeckle method is non-contact, non-invasive, and offers the possibility of conducting real-term measurements. Our measurement method could complement the existing tools as a measure to detect the effects due to a few minutes of exposure to FR. Investigations conducted on the healthiness of the plants and the age of the plants revealed that there was faster decay of the speckle correlation or a steep increase in BA in a healthy plant in comparison to an unhealthy plant of almost the same age, and when comparing age, a 1-month-old plant was found to have a faster decay of the correlation and thus a steep increase in BA in comparison to that of a 3-month-old plant. These results indicate that for unhealthy or aged plants, there is a slowing down of activity within the plant. Thus, based on our results, we demonstrated that our method has significant sensitivity, differentiating the response of the plant within minutes after exposure to FR or FR plus R. Although the mechanism remains elusive because of the complexity of the plant tissue, our method can work as a complementary measurement technique for speedier investigations of FR effects on plants.

## Data Availability

The raw data supporting the conclusions of this article will be made available by the authors, without undue reservation.

## Supplementary material

Supplementary Figure 1 BA as a function of time obtained in three sets with each lasting a period of 20s after exposure of the plant to FR with FR exposure time of 120 s. Here, the data were not acquired continuously for a minute but in three sets of 20 s each. Correlation and thus BA were obtained for each of the 20 s with the top representing the BA results obtained with speckles obtained within the 1^st^ 20 s. The middle and bottom represent the results of BA obtained with speckles in the set 2 corresponding to 20 to 40 s and the third set corresponding to 40 s to 60 s times of recording, respectively. Large changes can be seen in the first 20 s and so the data analysis was restricted to the first set of 20 s.

Supplementary Figure 2 BA as a function of time obtained for 60 s after exposure of the plant to FR with FR exposure time of 300 s. Here, the data were not acquired continuously for a minute but in three sets with each set lasting 20 s. The top represents the BA results obtained with speckles obtained within the 1^st^ 20 s. The middle and bottom represent the results of BA obtained with speckles in the 20s to 40 s and 40 s to 60 s times of recording, respectively. Although large changes are seen during all the windows of acquisition, in order to conduct a comparison with the results of 120-s exposure, the data analysis was restricted to the first 20 s.

Supplementary Information S3 Supplementary video under No FR for deadleaf.

Supplementary Information S4 Video under NoFR for a 1-month-old leaf.

Supplementary Information S5 Video under FR for a 1-month-old leaf.
