# Supplementary material for: Laser biospeckle as a method to investigate the short-term effects of far-red light on an arugula (Eruca sativa Mill) plant
Source: Front Plant Sci. 2025 Feb 25;16:1496790. doi: 10.3389/fpls.2025.1496790 (PMC11893838; doi:10.3389/fpls.2025.1496790)

Supplementary information S1

Figure S1. BA as a function of time obtained in three sets with each lasting over a period of 20s after exposure of the plant to FR with FR exposure time of 120 s. Here, the data were not acquired continuously for a minute but in three sets of 20 s each. Correlation and thus BA obtained for each of the 20 s with the top representing the BA results obtained with speckles obtained within the 1st 20 s. Middle and bottom represent respectively the results of BA obtained with speckles in the set 2 corresponding to 20 to 40 s and the third set corresponding to 40 s to 60 s times of recording. As can be seen large changes are seen with the first 20 s and so the data analysis was restricted to the first set of 20 s in the subsequent analysis.

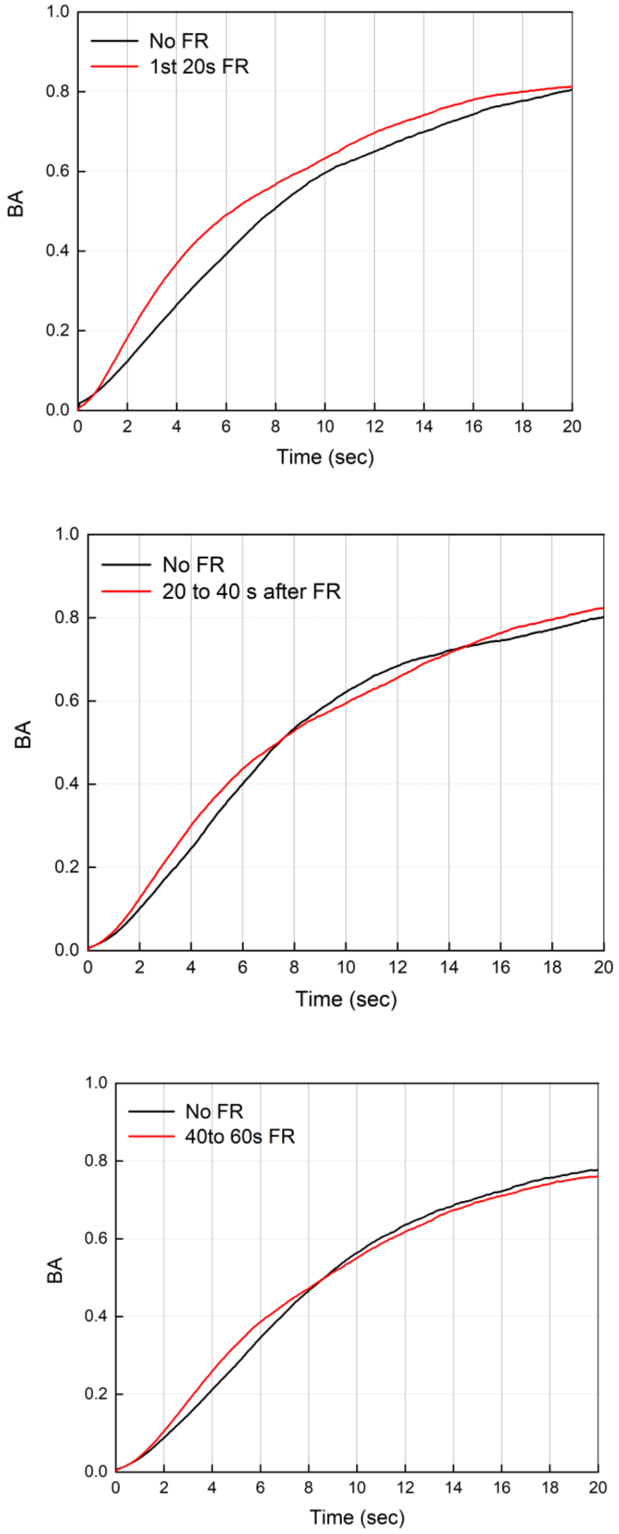

Supplement: Supplementary Figure 1 — BA as a function of time obtained in three sets with each lasting a period of 20s after exposure of the plant to FR with FR exposure time of 120 s. Here, the data were not acquired continuously for a minute but in three sets of 20 s each. Correlation and thus BA were obtained for each of the 20 s with the top representing the BA results obtained with speckles obtained within the 1st 20 s. The middle and bottom represent the results of BA obtained with speckles in the set 2 corresponding to 20 to 40 s and the third set corresponding to 40 s to 60 s times of recording, respectively. Large changes can be seen in the first 20 s and so the data analysis was restricted to the first set of 20 s. [file DataSheet1.pdf]
