# Supplementary material for: Laser biospeckle as a method to investigate the short-term effects of far-red light on an arugula (Eruca sativa Mill) plant
Source: Front Plant Sci. 2025 Feb 25;16:1496790. doi: 10.3389/fpls.2025.1496790 (PMC11893838; doi:10.3389/fpls.2025.1496790)
Supplement: Supplementary Figure 2 — BA as a function of time obtained for 60 s after exposure of the plant to FR with FR exposure time of 300 s. Here, the data were not acquired continuously for a minute but in three sets with each set lasting 20 s. The top represents the BA results obtained with speckles obtained within the 1st 20 s. The middle and bottom represent the results of BA obtained with speckles in the 20s to 40 s and 40 s to 60 s times of recording, respectively. Although large changes are seen during all the windows of acquisition, in order to conduct a comparison with the results of 120-s exposure, the data analysis was restricted to the first 20 s. [file DataSheet2.pdf]

Supplementary information S2

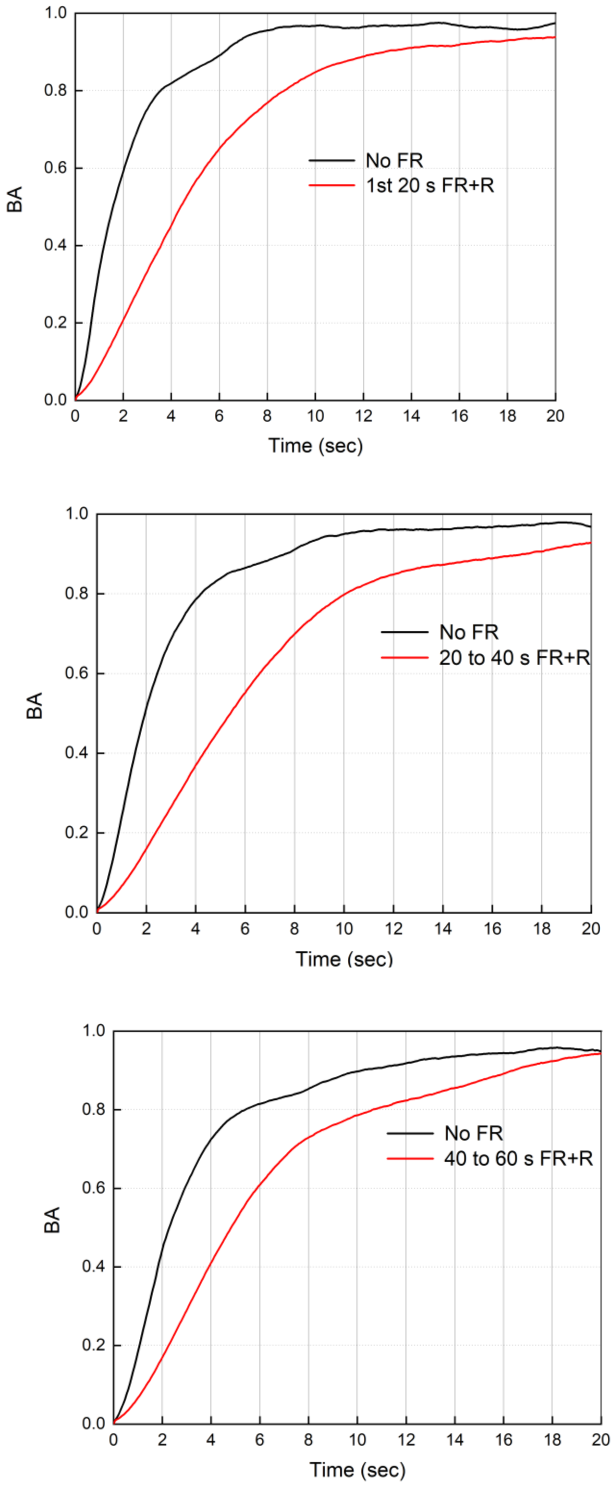

Figure S2. BA as a function of time obtained for 60 s after exposure of the plant to FR with FR exposure time of 300 s. Here, the data were not acquired continuously for a minute but in three sets with each set lasting 20 s. Results of BA with the top representing the BA results obtained with speckles obtained within the 1st 20 s. Middle and bottom represent respectively the results of BA obtained with speckles in the 20s to 40 s and 40 s to 60 s times of recording. Although large changes are seen during all the windows of acquisition, in order to do comparison with the results of 120s, the data analysis was restricted to the first 20 s in the subsequent analysis.
